# Assessment of Measurement Reliability for the IPN Test in Cardiac Patients

**DOI:** 10.3390/jcm9051552

**Published:** 2020-05-21

**Authors:** Łukasz Oleksy, Agnieszka Skiba, Iwona Sulowska, Marcin Trębowicz, Bartosz Rukasz, Artur Stolarczyk, Joanna Zyznawska, Anna Mika

**Affiliations:** 1 Physiotherapy and Sports Centre, Rzeszow University of Technology, 35-959 Rzeszow, Poland; loleksy@oleksy-fizjoterapia.pl; 2 Oleksy Medical & Sports Sciences, 37-100 Łańcut, Poland; 3 Institute of Clinical Rehabilitation, University of Physical Education in Krakow, 31-571 Krakow, Poland; agnieszka.skiba@awf.krakow.pl (A.S.); anna.mika@awf.krakow.pl (A.M.); 4 Maria Curie-Sklodowska University Branch in Puławy, 24-100 Puławy, Poland; Marcin.Trebowicz@poczta.umcs.lublin.pl; 5 “Werandki” Diagnostics & Physiotheraphy Centre, 24-140 Nałęczów, Poland; 6 Military Clinical Hospital with Outpatient Clinic, 20-049 Lublin, Poland; bartek.rukasz@gmail.com; 7 Orthopaedic and Rehabilitation Department, Medical University of Warsaw, 02-091 Warsaw, Poland; drstolarczyk@gmail.com; 8 Faculty of Health Sciences, Department of Physiotherapy, Jagiellonian University Medical College Krakow, 31-008 Krakow, Poland; joanna.zyznawska@uj.edu.pl

**Keywords:** IPN test, cardiac rehabilitation, submaximal tests, performance test

## Abstract

Cardiological diagnostics use maximal and submaximal tests with increasing load. Maximal stress tests are currently considered the gold standard. The Institut für Prävention und Nachsorge, Cologne (IPN) test may be an alternative when maximal patient load is not indicated. The universality of the test is well-documented in sport, but the reliability of this test is unknown. The aim of this study was to assess between-trial and between-day reliability for parameters assessed by the IPN stress test in cardiological patients.: In a study of 24 patients aged 39 to 79 years with cardiovascular diseases, the IPN cycle ergometer short test was performed (submaximal performance test). The reliability of heart rate, systolic and diastolic pressure, absolute power at submaximal load, relative performance at submaximal load and target heart rate were assessed. Good (Interclass Correlation Coefficient (ICC) values ranged from 0.832 to 0.894) and excellent (ICC values ranged from 0.904 to 0.969) between-trial reliability was noted. Between-day reliability was good (ICC values from 0.777 to 0.895) and excellent (ICC values from 0.922 to 0.950). The obtained results suggest that the IPN test may be a reliable tool for use in the assessment of cardiological patients, avoiding the implementation of maximal efforts when excessive patient load is not recommended.

## 1. Introduction

Cardiac rehabilitation plays an important role in the treatment of cardiovascular diseases. It has been shown that patients, following myocardial infarction and heart failure, live longer as a result of therapeutic treatment. Evaluating the impact of cardiac rehabilitation as an indispensable element in the rehabilitation of patients with cardiovascular diseases has been the subject of numerous studies [1,2,3,4,5,6,7]. Anderson et al. [4] published a systematic Cochrane review of cardiac rehabilitation, including 63 trials with 14,486 randomised participants. In the review, it was indicated that exercise-based cardiac rehabilitation reduces the risk of cardiovascular mortality in comparison to the no-exercise controls.

The basic method used in the rehabilitation of cardiological patients is endurance training. There are different ways to determine training intensity. One of them is assessment based on a patient’s subjective fatigue. This is a highly subjective and ineffective test in patients with concomitant disease reducing respiratory function. Another method is the cardiopulmonary exercise test (ergospirometry), a valuable evaluation tool allowing us to differentiate between the causes of dyspnoea in patients undergoing rehabilitation. Knowledge of characteristic changes in respiratory parameters observed during a stress test facilitates making clinical decisions and planning kinesiotherapy. Training intensity is also determined by means of stress tests performed on a treadmill or cycloergometer, which focus on electrocardiography. The assessment of the body’s response during physical effort is one of the most important elements of diagnostics used in cardiac rehabilitation. Some disease symptoms, such as, e.g., indications of myocardial ischaemia, early signs of failure or arrhythmia, can only manifest themselves when the body is under stress. The assessment of exercise tolerance is an essential element of programming the intensity of exercises, verifying the effectiveness of rehabilitation and assessing its progress. Testing is also useful in the qualification of the performance of certain professional activities or determining the intensity of physical activity [8,9].

By definition, cardiological diagnostics use maximal and submaximal tests with increasing loads, most often performed on an ergometer. Maximal stress tests are currently considered the gold standard. These tests are performed on a treadmill or cycloergometer and they determine the intensity of endurance physical training. In the rehabilitation of patients with cardiovascular diseases, the basic method applied in these cases is endurance physical training. The intensity of this training is determined on the basis of a maximal stress test on a treadmill or cycloergometer. In practice, several popular exercise tests are used to assess load. Physical exercise tolerance tests (physical work capacity (PWC)) comprise a series of trials performed on a cycloergometer. These graded load tests are used to determine performance at different heart rates, the parameters of which are set according to age and physical condition as well as individual characteristics and physical limitations. The tests are performed at different borderline heart rate values—from 130 to 170 beats/minute, depending on the preferred test. The test is carried out without intervals until reaching target heart rate. Usually, the gradual increase in load begins in accordance with WHO recommendations from 25–50 Watts, which should be increased every 2 min. by 25 Watts. Alternatively, the Hellmann/Venrath (HV) method of increasing the load can be used. The initial load of 30 W is increased every 3 min. by 40 watts [10,11]. Another method is the treadmill-based Bruce protocol, which starts at a speed of 2.74 km/h, while the grade is gradually increased by 10% every 3 min. [12]. For patients with limited exercise tolerance, the modified Bruce protocol is used. This test consists of two warm-up stages, each lasting 3 min. At 3-minute intervals, the speed and grade of the treadmill are increased by 2% [13]. There are also maximum permitted load tests (PWCmax) that rely on testing until reaching extreme fatigue; however, these types of tests are not applicable in the case of cardiac patients. The selection of an appropriate test and its course is necessary in cardiological diagnostics to determine individual training heart rate (HR) for basic endurance training and safe exercise [10,11].

One of the tests used to assess performance is the Institut für Prävention und Nachsorge, Cologne (IPN) test, which is a modification of the two- and four-stage tests according to Lagerstrom. The essence of the test is that there is no need to apply a maximal load during testing. It is carried out considering a subject’s individual parameters, such as age, sex, body mass, frequency of training and resting heart rate. It also takes the individual purpose of the test into account (rehabilitation, prophylaxis, recreational training). Fitness is measured in Watt/kg of body mass without achieving maximal fatigue. The obtained efficiency is compared to values in a table. The test results form the basis for determining training heart rate. The IPN test allows us to calculate criteria for applying intervals, determine load pattern, compare results with norms and obtain consistent results as well as training recommendations [11,14,15]. In comparative research monitoring lactate levels, the reliability of the IPN test has been confirmed, but this test is not a substitute for other comprehensive tests, e.g., ergospirometry [16]. The IPN test is widely used in fitness and planning training loads for athletes, but in the literature on the subject, there is a lack of research confirming its effectiveness in rehabilitation [17,18]. There are few literature items presenting the use of the IPN test to assess endurance capacity, for example, in children with haemophilia [19]. There are no reports on the application of the IPN test in the rehabilitation of cardiovascular patients. On the other hand, there are studies in which it is reported that maximal stress testing, when properly supervised, is safe for patients beginning cardiovascular rehabilitation [2,20,21]. However, if a test at maximal stress condition is not indicated, there is a need to choose a submaximal testing condition, which should be safe, valid and provide a reliable diagnosis.

The universality of the IPN test is well documented in sport, but the reliability of this test is still unknown. As we underlined, the safety of the IPN test, due to the lack of the requirement to achieve maximal fatigue, makes this test an alternative when maximal patient load is not indicated. Thus, this test is appropriate for patients with many health restrictions. Moreover, there are no reports on the use of the IPN test among cardiac patients or on the assessment of its reliability. The novelty of our study is that, for the first time, this test is validated.

The aim of the study is to assess between-trial and between-day reliability for parameters assessed by the IPN stress test in cardiological patients. As cardiac rehabilitation is an effective element in the treatment of cardiovascular diseases, the implementation of an easy-to-use and effective tool for assessing patient stress would significantly reduce time and facilitate diagnostics with benefits for patients.

## 2. Experimental Section

### 2.1. Study Group

The study group consisted of 24 adults (13 women and 11 men) aged 39 to 79, diagnosed with cardiovascular disease (chronic ischemic heart disease, stable hypertension, condition after aortic valve implantation). All patients included in the study were in at a chronic stage of the disease which allowed for the implementation of moderate physical exercise. The tests were conducted while the patients were at a health resort for a rehabilitation stay. During admission to the rehabilitation centre, all patients were examined by a cardiologist and none of them who had the contraindications for physical exercises were allowed to participate in the rehabilitation stay. The characteristics of the study group are provided in Table 1.

The inclusion criteria were:

- diagnosed chronic cardiovascular diseases (2nd degree according to NYHA—New York Heart Association);
- no health contraindications to perform physical exercise;
- written consent of the subject to participate in the study;
- consent of the doctor supervising the project to participate in the research.

The exclusion criteria were:

- heart insufficiency, instable angina pectoris, severe vessel diseases, pulmonary embolism, thrombosis of large vessels, cerebral ischemia, liver and kidney disorders, acute inflammation and/or diabetes mellitus.

All participants were informed of the research protocol in detail and provided their written informed consent to participate in the study, which was approved by the Ethical Committee (18/KBL/OIL/2016). All procedures were performed in accordance with the 1964 Helsinki declaration and its later amendments.

### 2.2. Testing Methods

From the moment the study began, qualified subjects were required to abstain from other forms of exercise therapy for the duration of the experiment. Before beginning research, the participants were familiarised with information on the purpose of the study, the applied methodology and the possibility of resigning from further participation in the project at any stage of its duration. The qualified individuals were tested twice on the same day to check between-trial-reliability, which was also subjected to validation. The tests were carried out 1–2 days apart, under the same conditions and at the same time of day.

### 2.3. Testing Procedure

Before testing began, a survey was conducted to obtain information on patients’ declared physical activity. Then, resting heart rate was measured and, on this basis, individual, baseline ergometer settings were appropriated (target heart rate was determined during the IPN test). The resting HR value was obtained during the week prior to the test. HR was measured by a nurse in the morning, directly after waking up, while the patients still laid in bed. Then, the values from 5–6 days were averaged. The test was performed by the Kardiomed 700 Ergometer (PROXOMED MEDIZINTECHNIK GMBH) device equipped with heart rate monitoring function.

According to the recommendations of the IPN test, the target heart rate was determined on the basis of resting heart rate and age of the subjects. Then, with reference to the daily physical activity of patients (training frequency, type of activity), target heart rate was adjusted taking the classification of activities into account. Level 1 (definitions of cardiovascular endurance training level —1 not trained (no cardio vascular training)) was selected for the examined patients in the IPN test protocol.

One by one, the load scheme appropriate for the patient’s condition was then selected. The World Health Organization (WHO) scheme (25 Watts start load, increased by 25 Watts every 2 min.) or Hellmann/Venrath (30 Watts start load, increased by 40 Watts every 3 min.) were used. The final heart rate was determined by the program based on standard reference data. The HV scheme was used when load limits were minimal. The WHO method was applied for lower values. Each patient was tested until reaching target heart rate. The achieved efficacy (Watts) was recorded and divided by the subject’s body mass (Watts per kg of body mass BM). The efficacy value was automatically determined with the use of software. The obtained efficiency values were compared with reference data. Based on the achieved values, the load index and training heart rate for aerobic endurance training were obtained.

In the examined group, the IPN cycle ergometer short test was used (submaximal performance test). Baseline results were calculated by entering age, body mass, activity level and the presence of risk factors—the program itself calculated which protocol was applied to grade the load and continuously performed selection on a regular basis using both WHO and Hollman-Venrath (HV). A constant element was that these were the patients with the lowest level of fitness (level 1) and the IPN 1 test was chosen. The remaining loads were selected based on age, risk factors from the interview and the patient’s body mass.

Analysed parameters:

Heart rate (BMP)—value of heart rate measurement before test;
Systolic pressure (pre) (mmHg)—value of systolic pressure measurement before test;
Diastolic pressure (pre) (mmHg)—value of the diastolic pressure measurement before test;
Heart rate (post) (BMP)—value of heart rate measurement after test;
Systolic pressure (post) (mmHg)—value of systolic pressure measurement after test;
Diastolic pressure (post) (mmHg)—value of the diastolic pressure measurement after test;
Absolute power at submaximal load (Watts);
Relative performance at submaximal load (Watts/kg);
Target HR (BMP).

Heart rate values at subsequent test stages (BMP):

RECOM. FROM/ RECOM. TO—from 79 to 96, aerobic exercise (regeneration/compensation zone);
BE1 FROM/ BE1 TO—from 96 to 107, aerobic exercise (basic strength 1);
BE2 FROM/ BE2 TO—from 107 to 119, aerobic exercise (basic endurance 2);
DA FROM/ DA TO—from 119 to 130, aerobic exercise (area of rehabilitation);
CS—heart rate > 137—anaerobic exercise (area of competition).

### 2.4. Statistical Analysis

Statistical analysis was carried out using STATISTICA 12.0 software. To assess the normality of variable distribution, the Shapiro–Wilk test was performed. The between-trial and between-day reliability of the sEMG variables were determined using Intraclass Correlation Coefficients (ICC) [22]. The interpretation of the ICC agreement was performed according to Koo et al. [23]: below 0.50—poor; between 0.50 and 0.75—moderate; between 0.75 and 0.90—good; above 0.90—excellent. The variability within each data set was described using coefficients of variation (CV), based on the mean and SD values. Additionally, in order to compare the results of the authors’ research with the reliability presented by other authors, Pearson’s linear correlation coefficient (r) was calculated for both between-trial and between-day comparisons. The two-tailed level of statistical significance was set at *p* < 0.05.

## 3. Results

### 3.1. IPN Test between-Trial-Reliability

The IPN test’s between-trial reliability may be assessed as good (0.832–0.894 ICC) and excellent (0.904–0.969 ICC) (Table 2). Strong correlations were noted between measurements (r = 0.722–0.858; *p* = 0.001). Heart rate measurements 1 and 2 were very strongly correlated (r = 0.942; *p* = 0.001). A moderate correlation was only observed for the basic endurance 2 (BE2) FROM parameter in measurements 1 and 2 (r = 0.545). All correlations were statistically significant. The coefficient of variation in measurements 1 and 2 did not show high variation across all parameters, with its value ranging from 7.13 to 18% (Table 2).

### 3.2. IPN Test between-Day Reliability

The between-day reliability of the assessed parameters was good (0.777–0.895 ICC) and excellent (0.922–0.950 ICC) (Table 3). Strong correlations (r = 0.731–0.861; *p* = 0.001) were observed for the majority of parameters. There were moderate correlations between heart rate and diastolic parameters between days (r = 0.638–0.679; *p* = 0.01). All of the noted correlations were statistically significant. The coefficient of variation in measurements 1 and 3 showed little variation across all parameters, ranging from 4.86 to 17.42% (Table 3).

## 4. Discussion

The aim of this study was to evaluate parameter reliability assessed via the IPN test in cardiac patients. This is the first work evaluating IPN test reliability. Research regarding this test to date is sparse and is focused on fitness evaluation rather than reliability [15,17].

Physical training is an indispensable element of treatment among patients with ischemic heart disease, left ventricular failure or following heart transplantation or surgery. Nonetheless, planning rehabilitation programmes for cardiological patients is difficult and time-consuming as the physical exercise load must be individually adapted to each patient. In the case of these patients, rehabilitation should be programmed by taking the knowledge of proper heart rate values into account, thereby avoiding improper, often harmful loads. Proper implementation leads to a gradual increase in physical fitness [8,24,25].

In cardiac patients, a maximal stress test (the gold standard in the case of healthy individuals) may be performed in the absence of contraindications and with an adequate ECG control [26]. In the literature, it has been reported that submaximal tests are also efficient in assessing patients’ functional limitations and effectiveness of the applied therapy [27,28,29]. Based on the authors’ results, it has been suggested that the IPN test may be useful for cardiac patient assessment, allowing us to avoid maximal load application. The safety of these patients should be ensured by selecting a properly verified test, thus, a crucial element in test diagnostic value concerns its reliability, around which this study was focused.

One of the submaximal tests often used in cardiac patients is the 6-Minute Walk Test (6-MWT). It is a simple and safe test for assessing functional fitness. Its reliability was evaluated by Kervio et al. [30]. In addition, they compared the results of this tool with the maximal treadmill stress test. Their study was carried out among healthy people aged 60–70, without chronic diseases. The subjects performed the maximal stress test twice and the 6-Minute Walk Test five times—twice in the morning and three times in the afternoon. The distance covered during 6 min. increased with each attempt, indicating the learning effect. These differences were smaller after two attempts to perform the test. However, the coefficient of variation was constantly lower than 6%, indicating high test reliability. A strong correlation was found between the results of the 6-MWT and VO2max measured during the maximal stress test. Exercise intensity during the 6-MWT was submaximal, averaging 79.6% of VO2max [30].

Another method used to estimate VO2max is the Astrand and Ryhming (A-R) Cycle Ergometer Test [31]. Astrand [32] assessed the correlation between VO2 max estimated on the basis of the submaximal test and the level of VO2max obtained during the maximal test. There was a moderate correlation between variables (r = 0.71). Other researchers rated the reproducibility of this test and correlation with VO2max, including older [33] or sedentary people [34] in their research. However, there are no studies in which test reproducibility is assessed in cardiac patients, probably because of numerous limitations making these tests impossible to perform in this population.

Ebbeling et al. [35] developed the Single-Stage Submaximal Treadmill Walking Test (S-STWT), consisting of a warm-up session and one stage proper on a treadmill. The study was conducted among healthy people aged 20 to 59 years. A strong correlation was obtained (r = 0.96). Due to the fact that the test consists of only one proper stage, the authors recommend its use in people with reduced performance. However, there are no reports regarding cardiac patients.

In available studies, the effectiveness of the IPN test in programming sports training is highlighted. It is also indicated that this test is a good alternative to expensive and complex diagnostic methods or lactate concentration tests [15,17]. The availability and automatic calculation of results may cause economic replacement for expensive and complex diagnostic methods. Nevertheless, the use of the IPN test as a replacement requires a reliability evaluation.

In another study among women and men aged 20–60, compliance was evaluated with regard to the determination of training recommendations using the IPN test. The obtained results were compared with lactate concentration measurements in a group of 52 subjects, achieving their compliance. In 92% of people exercising according to the recommended training heart rate, the obtained lactate levels were within limits of standard deviation [16].

The IPN test is carried out in stages to check the performance of various load levels. In addition, the IPN test allows us to monitor a patient’s efficacy status, thanks to the possibility of periodic repetition. Changes in frequency of activity, body mass or performance make it necessary to alter target heart rate or load pattern, thus, in this case, regular efficacy evaluation should be carried out approximately every 3 months.

In this study, the reliability of measurements was verified in successive exercise load zones. The reliability of heart rate values for aerobic exercise was tested in three zones: from 84 to 99 bpm, 91 to 111 bpm, 106 to 129 bpm and from 122 to 127 bpm. The majority of parameters achieved excellent reliability. The reproducibility of measurements obtained for anaerobic exercise was also assessed in cases of the load being above 137 bpm. Excellent reliability has also been shown for this parameter.

There are no available studies in which the reproducibility of the IPN test by assessing ICC measurement reliability has been confirmed. Other published reports concern the validity of exercise tests on an ergometer evaluated using predicted VO2peak. Research was carried out among a group of adults with atetospastic cerebral palsy. Predicted VO2peak assessment was performed using a submaximal cycloergometer test, while VO2peak was evaluated using the maximal stress test. It has been shown that a multistage submaximal ergometer test can provide correct information when estimating the performance of adults with atetospastic cerebral palsy [36].

There are a lot of people around the world suffering from serious cardio-pulmonary restrictions and/or many other health complications. Therefore, like regular cardiac patients (evaluated in this study), they require safe, adequate and individually tailored methods for general fitness assessment and rehabilitation. Therefore, the authors suggest the usefulness of the IPN test for these patients. The results obtained in this novel study, reporting the high reliability of the IPN test in cardiac patients confirms that the IPN test may now be safely and reliably used in clinical conditions.

### Study Limitations

The presented study is characterised by some limitations. The first is the small group size, totalling 24 subjects. In addition, the group was not homogeneous in terms of age—the youngest participant was 39 years old and the oldest 79. Because the IPN test is dedicated to patients with many health-related restrictions and a low endurance level as a result, the target workloads calculated via the IPN test are relatively low. Therefore, during the rehabilitation process, the level of training loads should be constantly monitored to avoid progress-limiting effects of low exercise intensity.

There is a need to validate the IPN test in other groups of patients with different health problems than cardiological issues, e.g., pulmonary, neurological or rheumatologic. In addition, there is a need to check the reliability of the IPN test in patients with a higher endurance level to make sure, that at higher effort intensity, the ICC values of assessed parameters still will be as high as those determined at low intensity.

Because the IPN test is easy to perform and safe, even for patients with a very low endurance level, it would be worth developing an IPN test for home use. The chest belt HR sensor and mobile application may be very useful in the monitoring of loads during daily physical activity in people with serious health restrictions.

## 5. Conclusions

In the authors’ study, the high reliability of the IPN stress test used in cardiac patients was shown. Good and excellent reliability was also demonstrated, both in between-trial and between-day cases. The obtained results suggest that the IPN test may be a reliable tool for use in the assessment of cardiological patients, avoiding the implementation of maximal efforts when excessive patient load is not recommended.

## Figures and Tables

**Table 1:** Characteristics of the study group.

Parameter	Women (n = 13) Mean ± SD	Men (n = 11) Mean ± SD
Age (years)	63.15 ± 8.62	66.73 ± 5.66
Body mass (kg)	76.00 ± 11.84	86.00 ± 16.43

SD—standard deviation.

**Table 2:** Institut für Prävention und Nachsorge, Cologne (IPN) test’s between-trial reliability.

Parameter	ICC	r (First (1) and Second (2) Measurement)	Mean 1	SD 1	CV(%) 1	Mean 2	SD 2	CV(%) 2
Heart rate (BMP)	0.883	0.791	72.17	11.94	16.55	72.83	12.00	16.48
Systolic pressure (pre) (mmHg)	0.894	0.814	133.96	17.52	13.08	132.96	15.45	11.62
Diastolic pressure (pre) (mmHg)	0.864	0.763	81.96	11.52	14.05	81.42	10.57	12.99
RECOM from (BMP)	0.966	0.854	84.94	3.70	7.29	84.24	4.40	8.12
RECOM to (BMP)	0.963	0.843	103.24	4.40	7.19	102.24	5.13	7.98
BE1 from (BMP)	0.963	0.843	103.24	4.40	7.19	102.24	5.13	7.98
BE1 to (BMP)	0.968	0.856	115.35	5.26	7.39	114.24	6.08	8.20
BE2 from (BMP)	0.832	0.545	115.35	5.26	7.39	117.18	10.25	9.72
BE2 to (BMP)	0.963	0.848	127.35	5.26	7.13	126.35	6.29	7.93
DA from (BMP)	0.963	0.848	127.35	5.26	7.13	126.35	6.29	7.93
DA to (BMP)	0.967	0.858	139.47	6.14	7.30	138.35	7.25	8.12
CS (BMP)	0.967	0.858	139.47	6.14	7.30	138.35	7.25	8.12
Heart rate (post) (BMP)	0.969	0.942	74.42	12.96	17.42	76.79	13.82	18.00
Systolic pressure (post) (mmHg)	0.839	0.722	133.17	15.96	11.99	138.50	16.13	11.64
Diastolic pressure (post) (mmHg)	0.904	0.827	81.83	10.35	12.65	83.38	11.17	13.40
Absolute power at submaximal load (Watt)	0.970	0.955	108.20	24.33	22.48	104.87	25.57	24.38
Relative performance at submaximal load (Watt/kg)	0.950	0.920	1.35	0.21	15.25	1.31	0.24	18.76
Target HR (BMP)	0.990	0.994	124.58	9.99	8.01	124.79	9.83	7.87

Intraclass Correlation Coefficients (ICC); Pearson’s linear correlation coefficient (r); standard deviation (SD); coefficient of variation (CV).

**Table 3:** IPN test between-day reliability.

Parameter	ICC	r (First (1) and Third (3) Measurement)	Mean 1	SD 1	CV(%) 1	Mean 3	SD 3	CV(%) 3
Heart rate (BPM)	0.777	0.638	72.17	11.94	16.55	74.13	11.02	14.86
Systolic pressure (pre) (mmHg)	0.860	0.731	133.96	17.52	13.08	132.38	13.65	10.31
Diastolic pressure (pre) (mmHg)	0.869	0.787	81.96	11.52	14.05	80.71	9.90	12.26
RECOM from (BPM)	0.885	0.804	84.94	3.70	7.29	84.53	4.36	5.09
RECOM to (BPM)	0.895	0.821	103.24	4.40	7.19	102.71	5.18	4.94
BE1 from (BPM)	0.895	0.821	103.24	4.40	7.19	102.71	5.18	4.94
BE1 to (BPM)	0.894	0.817	115.35	5.26	7.39	114.71	6.08	5.21
BE2 from (BPM)	0.894	0.817	115.35	5.26	7.39	114.71	6.08	5.21
BE2 to (BPM)	0.888	0.812	127.35	5.26	7.13	126.82	6.29	4.86
DA from (BPM)	0.888	0.812	127.35	5.26	7.13	126.82	6.29	4.86
DA to (BPM)	0.887	0.807	139.47	6.14	7.30	138.82	7.19	5.09
CS (BPM)	0.887	0.807	139.47	6.14	7.30	138.82	7.19	5.09
Heart rate (post) (BPM)	0.922	0.856	74.42	12.96	17.42	77.75	12.83	16.50
Systolic pressure (post) (mmHg)	0.925	0.861	133.17	15.96	11.99	135.67	16.87	12.44
Diastolic pressure (post) (mmHg)	0.950	0.679	81.83	10.35	12.65	81.58	9.89	12.12
Absolute power at submaximal load (Watt)	0.960	0.930	108.20	24.33	22.48	105.12	25.99	24.72
Relative performance at submaximal load (Watt/kg)	0.900	0.824	1.35	0.21	16.25	1.30	0.22	17,08
Target HR (BMP)	0.990	0.994	124.58	9.99	8.01	124.79	9.83	7.87

Intraclass Correlation Coefficients (ICC); Pearson’s linear correlation coefficient (r); standard deviation (SD); coefficient of variation (CV).

## References

[1] Fernhall B. (2013). Long-term aerobic exercise maintains peak VO2, improves quality of life, and reduces hospitalisations and mortality in patients with heart failure. J. Physiother..

[2] O’Connor C.M., Whellan D.J., Lee K.L., Keteyian S.J., Cooper L.S., Ellis S.J., Leifer E.S., Kraus W.E., Kitzman D.W., Blumenthal J.A. (2009). Efficacy and safety of exercise training in patients with chronic heart failure: HF-ACTION randomized controlled trial. JAMA.

[3] Piepoli M.F., Corrà U., Benzer W., Bjarnason-Wehrens B., Dendale P., Gaita D., McGee H., Mendes M., Niebauer J., Zwisler A.D. (2010). Secondary prevention through cardiac rehabilitation: From knowledge to implementation. A position paper from the Cardiac Rehabilitation Section of the European Association of Cardiovascular Prevention and Rehabilitation. Eur. J. Cardiovasc. Prev. Rehabil..

[4] Anderson L., Thompson D.R., Oldridge N., Zwisler A.D., Rees K., Martin N., Taylor R.S. (2016). Exercise-based cardiac rehabilitation for coronary heart disease. Cochrane Database Syst. Rev..

[5] Marchionni N., Fattirolli F., Fumagalli S., Oldridge N., Del Lungo F., Morosi L., Burgisser C., Masotti G. (2003). Improved exercise tolerance and quality of life with cardiac rehabilitation of older patients after myocardial infarction: Results of a randomized, controlled trial. Circulation.

[6] Oerkild B., Frederiksen M., Hansen J.F., Prescott E. (2012). Homebased cardiac rehabilitation is an attractive alternative to no cardiac rehabilitation for elderly patients with coronary heart disease: Results from a randomised clinical trial. BMJ Open.

[7] Anderson L., Sharp G.A., Norton R.J., Dalal H., Dean S.G., Jolly K., Cowie A., Zawada A., Taylor R.S. (2017). Home-based versus centre-based cardiac rehabilitation. Cochrane Database Syst. Rev..

[8] Fletcher G.F., Balady G.J., Amsterdam E.A., Chaitman B., Eckel R., Fleg J., Froelicher V.F., Leon A.S., Piña I.L., Rodney R. (2001). Exercise standards for testing and training: A statement for healthcare professionals from the American Heart Association. Circulation.

[9] Mezzani A., Corrà U., Giordano A., Cafagna M., Adriano E.P., Giannuzzi P. (2007). Unreliability of the %VO2 reserve versus % heart rate reserve relationship for aerobic effort relative intensity assessment in chronic heart failure patients on or off beta-blocking therapy. Eur. J. Cardiovasc. Prev. Rehabil..

[10] Alpert J.S. (2008). A Water-Based Exercise Program for patients with coronary artery disease. Cardiology.

[11] Trunz E. (1997). Der IPN-ausdauertest nach lagerstrøm-eine zusammenfassende darstellung als anleitung für den praxiseinsatz. Trainer.

[12] Bruce R.A., Kusumi F., Hosmer D. (1973). Maximal oxygen intake and nomographic assessment of functional aerobic impairment in cardiovascular disease. Am. Heart J..

[13] Bruce R.A. (1971). Exercise testing of patients with coronary heart disease. Principles and normal standards for evaluation. Ann. Clin. Res..

[14] Lagerstrøm D., Trunz E. (1997). IPN-Ausdauertest. Gesundh. Sport Ther..

[15] Trunz E., Lagerstrøm D., Giesen H., Ochs S. (2000). Der IPN-test teil 2. Bodylife.

[16] Herbort H. (1996). Überprüfung und Anwendung der Lagerstrøm-Formel zur Bestimmung der Trainingsherzfrequenz in Unterschiedlichen Test.-Situationen auf dem Fahrrad-Ergometer.

[17] Trunz E., Giesen H., Ochs S. (1999). Der IPN-test teil 1. Bodylife.

[18] Zeni A.I., Hoffman M.D., Clifford P.S. (1996). Energy expenditure with indoor exercise Machines. JAMA.

[19] Seuser A., Boehm P., Ochs S., Trunz-Carlisi E., Halimeh S., Klamroth R. (2015). How fit are children and adolescents with haemophilia in Germany? Results of a prospective study assessing the sport-specific motor performance by means of modern test procedures of sports science. Haemophilia.

[20] Balady G.J., Arena R., Sietsema K., Myers J., Coke L., Fletcher G.F., Forman D., Franklin B., Guazzi M., Gulati M. (2010). Clinician’s Guide to cardiopulmonary exercise testing in adults: A scientific statement from the American Heart Association. Circulation.

[21] Keteyian S.J., Isaac D., Thadani U., Roy B.A., Bensimhon D.R., McKelvie R., Russell S.D., Hellkamp A.S., Kraus W.E. (2009). HF-ACTION Investigators. Safety of symptom-limited cardiopulmonary exercise testing in patients with chronic heart failure due to severe left ventricular systolic dysfunction. Am. Heart J..

[22] Shrout P.E., Fleiss J.L. (1979). Intraclass correlations: Uses in assessing rater reliability. Psychol. Bull..

[23] Koo T.K., Li M.Y. (2016). A guideline of selecting and reporting intraclass correlation coefficients for reliability research. J. Chiropr. Med..

[24] Roger V.L., Jacobsen S.J., Pellikka P.A., Miller T.D., Bailey K.R., Gersh B.J. (1998). Prognostic value of treadmill exercise testing. A population-based study in Olmsted County, Minnesota. Circulation.

[25] Snader C.E., Marwick T.H., Pashkow F.J., Harvey S.A., Thomas J.D., Lauer M.S. (1997). Importance of estimated functional capacity as a predictor of all-cause mortality among patients referred for exercise thallium single-photon emission computed tomography: Report of 3400 patients from a single center. J. Am. Coll. Cardiol..

[26] Noonan V., Dean E. (2000). Submaximal exercise testing: Clinical application and interpretation. Phys. Ther..

[27] Hagberg J.M. (1994). Exercise assessment of arthritic and elderly individuals. Baillieres Clin. Rheumatol..

[28] Marciniuk D.D., Gallagher C.G. (1994). Clinical exercise testing in interstitial lung disease. Clin. Chest. Med..

[29] Questead K.A., Alquist A. (1994). Exercise assessment in clinical practice. Phys. Med. Rehabil. Clin. N. Am..

[30] Kervio G., Carre F., Ville N.S. (2003). Reliability and intensity of the six-minute walk test in healthy elderly subjects. Med. Sci. Sports Exerc..

[31] Astrand P.O., Ryhming I. (1954). A nomogram for calculation of aerobic capacity (physical fitness) from pulse rate during submaximal work. J. Appl. Physiol..

[32] Astrand I. (1960). Aerobic capacity in men and women with special reference to age. ACTA Physiol. Scand. Suppl..

[33] Hartung G.H., Blancq R.J., Lally D.A., Krock L.P. (1995). Estimation of aerobic capacity from submaximal cycle ergometry in women. Med. Sci. Sports Exerc..

[34] Teraslinna P., Ismail A.H., MacLeod D.F. (1966). Nomogram by Astrand and Ryhming as a predictor of maximum oxygen intake. J. Appl. Physiol..

[35] Ebbeling C.B., Ward A., Puleo E.M., Widrick J., Rippe J.M. (1991). Development of a single-stage submaximal walking test. Med. Sci. Sports Exerc..

[36] Satonaka A., Suzuki N., Kawamura M. (2012). Validity of submaximal exercise testing in adults with athetospastic cerebral palsy. Arch. Phys. Med. Rehabil..

